# In-hospital and long-term mortality after venoarterial ECMO for refractory cardiogenic shock

**DOI:** 10.1186/cc14361

**Published:** 2015-03-16

**Authors:** M Bottiroli, T Maraffi, D Decaria, S Nonini, E Montrasio, K Gervasio, F Milazzo, R Paino

**Affiliations:** 1 Anestesia Rianimazione 3, A.O. Niguarda, Milan, Italy

## Introduction

Venoarterial (VA) ECMO is used for mechanical support in patients with cardiogenic shock (CS) unresponsive to medical therapy. Long-term survival and quality of life after hospital discharge have not yet been well analyzed.

## Methods

We performed a retrospective observational study of patients admitted to the ICU for refractory CS from January 2010 to November 2014. Patients with postcardiotomy and/or post-transplant CS were excluded. Demographic, clinical and biochemical variables were collected. Continuous variables are presented as mean (standard deviation) and categorical variables as percentage. Long-term outcome and quality of life were assessed during scheduled follow-up evaluations or telephonic interviews.

## Results

We analyzed 23 consecutive patients undergoing VA ECMO for refractory CS. Etiologies of cardiac collapse were: 11 acute myocarditis, five acute myocardial infarctions and seven acute decompensation of chronic cardiomyopathies (CCM). Thirteen patients died during the hospital stay and 10 survived. The main cause of ICU death was progressive multiple organ dysfunction (12/13). Baseline variables are presented in Table 1. All patients discharged from the hospital are still alive at follow-up (median 27 months, range 4 to 56) with a median NYHA class of 1 (range 1 to 2). All patients except one returned to an active style of life. Multivariate analysis (Cox) revealed pre-ECMO SOFA score (HR = 2.18, 95% CI = 1.016 to 4.6) and history of CCM (HR = 19, 95% CI = 2 to 178) and pre-ECMO lactate (HR = 1.2, 95% CI = 1.02 to 1.4) as independent risk factors for hospital mortality.

**Table 1:** 

	Alive	Dead	*P *value
Age (years)	30 ± 18	43 ± 14	0.012
CCM	0 (0%)	7 (100%)	0.014
SOFA	8 ± 1.4	10 ± 1.9	0.002
Creatinine (mg/dl)	1.1 ± 0.3	2.2 ± 0.7	0.001
Bilirubin (mg/dl)	1 ± 0.4	2 ± 1.8	0.09
Lactate (mmol/l)	5 ± 2.1	8 ± 5.3	0.021
Platelets (103/μl)	257 ± 79	162 ± 99	0.03

## Conclusion

VA-ECMO is an effective treatment tool for refractory CS in patients with acute life-threatening heart failure. Patients affected by acute decompensation of CCM had poorer outcomes characterized by multiple organ dysfunction, as already known in the literature.

